# Dynamical and Stochastic Analysis of a Piezoelectric Neuron Model for Intelligent Sensing Applications

**DOI:** 10.3390/s26103179

**Published:** 2026-05-17

**Authors:** Atef Abdelkader, Haiqa Ehsan, Adil Jhangeer

**Affiliations:** 1 College of Humanities and Sciences, Ajman University, Ajman P.O. Box 346, United Arab Emirates; 2 IT4Innovations, VSB—Technical University of Ostrava, 70800 Ostrava, Czech Republic; adil.jhangeer@vsb.cz; 3 Faculty of Electrical Engineering and Computer Science, VSB—Technical University of Ostrava, 70800 Ostrava, Czech Republic; 4 Center for Theoretical Physics, Khazar University, 41 Mehseti Str., Baku AZ1096, Azerbaijan; 5 Department of Computer Engineering, Biruni University, Istanbul 34015, Turkey

**Keywords:** neuronsystem, dynamical system, basin of attraction, stochastic analysis, probability density distribution, recurrence plot

## Abstract

In this work, we explore a piezoelectric neuron model in deterministic perturbations and stochastic forcing due to its use in mechanically driven sensing systems and neuromorphic sensor design. The model comprises of fast activation and slow recovery behaviors and constitutes a multiscale excitable system, converting external mechanical perturbations into nonlinear electrical responses. We initially examine the deterministic dynamics with phase-space reconstruction, basin of attraction mapping, return map analysis and sensitivity to initial conditions. These findings demonstrate stable limit-cycle oscillations and high nonlinear sensitivity that are crucial to high-resolution sensing and signal amplification. Stochastic forcing is added in order to include realistic environmental effects, and solved numerically with the Euler-Maruyama scheme. Time-series statistics, phase portraits, and recurrence quantification analysis are used to analyze the resulting ensemble dynamics, making it possible to characterize the variability and loss of predictability caused by noise. Comparison of deterministic and stochastic regimes indicates that the intensity of noise can considerably alter the firing patterns and recurrence structures.

## 1. Introduction

One of the most complex and highly nonlinear systems in nature is the nervous system, making it difficult to study experimentally and challenging to describe theoretically. Mathematical models describe the dynamics of the signal generation, transmission, and processing performed by neurons, and address the multiscale nature, nonlinearity, and stochasticity of neural dynamics [1,2]. In time, computational neuroscience has emerged as a vital interdisciplinary science that integrates biology, physics and mathematics and allows to analyze brain dynamics beyond the scope of purely experimental methods [3]. The Hodgkin-Huxley model [4] and the simplified FitzHugh-Nagumo model [5] are classical neuron models that were used to explain the excitability and generation of action potential in neurons. The models that followed incorporated the significant idea of fast/slow dynamics: fast activation variables act upon slow recovery variables, resulting in phenomena of oscillations, excitability and bursting [6,7]. This framework gave an initial framework of how to think about neural signal processing as a nonlinear dynamical system. This was extended further by the Hindmarsh-Rose model [8] which could reproduce more complex forms of neuronal activity like bursting and chaotic dynamics. This model increased the link between the theory of nonlinear dynamical systems and neuronal biophysics, showing the feasibility of modelling rich and realistic neural activity patterns using the help of mathematical models. The Piezoelectric materials have a special kind of electromechanical transduction properties and have been the focus of much research and development of the scientific and engineering community, as they can convert mechanical stress into electrical potential or vice versa [9]. Quartz, barium titanate and lead zirconate titanate are some materials with high electromechanical coupling and are commonly employed for sensors, actuators and energy harvesting devices  [10,11].

The discovery of piezoelectricity was made by Jacques and Pierre Curie in 1880 [12] and since then has been widely used in precision instrumentation, and has been recently introduced to biomedical and neuromorphic engineering fields. The piezoelectric transducers are able to sense mechanical vibration, such as acoustic waves, and output electrical signals, and they have a functional analogy to mechanotransduction in the biological sense such as the cochlea and auditory nerve pathway [13]. This similarity has stimulated the creation of models based on biological systems that correlate mechanical sensing with neural-like processing. A model of this kind is the piezoelectric neuron system, PNS, which simulates the auditory transduction pathway (ATP) in which mechanical vibrations are converted into electrical signals sending to neural-like firing dynamics [14]. The model mimics one of the most important properties of the biological ear, which is that sound waves cause vibrations of the eardrum, followed by mechanical amplification and neural signaling by the hair cells of the cochlea [15]. The PNS provides a method for studying mechanical-electrical interactions between electrically-excitable dynamics and non-linear circuits that are coupled to a piezoelectric transducer with FitzHugh-Nagumo dynamics [16,17]. Combining mechanical energy transduction with nonlinear neural modeling offers an exciting paradigm for future compact, energy efficient, mechanosensitive neuromorphic hardware [18]. The applications of piezoelectric neural circuits, noise-induced nonlinear phenomena, in neural engineering, signal processing and bio-inspired electronic systems, have attracted attention in recent years. Stochastic perturbations have been shown to have a constructive effect in nonlinear excitable systems by improving the signal regularity and synchronization by coherence resonance in several studies. Recent success has also been realized in the field of memristive neuron circuits, piezoelectric energy coupled neural systems, and nonlinear resonance mechanisms in excitable dynamics. Inspired by these advances, the present study examines the dynamical behavior of a piezoelectric neuron circuit and looks into the appearance of coherence resonance under external perturbations.

Piezoelectric neuron models are at the interface of nonlinear dynamics, materials science, and computational neuroscience. The PNS is also based on a physical transduction process that correlates mechanical stimuli with electrical neural responses, unlike purely abstract neuron models, and has applications to artificial sensory systems and neuromorphic devices [19]. Recent research has shown that piezoelectric ceramics in combination with FHN circuits could be used to simulate a wide variety of neural behaviors such as synchronization, noise-induced firing and chaotic behavior with synaptic and time-delayed feedback-coupled behavior [20]. In addition, ultrasound-activated piezoelectric nanoparticles have been demonstrated to induce neurostem cell differentiation by calcium signaling pathways, which are capable of providing transformative regenerative uses of neuroscience [21]. The nonlinear behavior of piezoelectric neuron models is especially fruitful as a result of interaction between the mechanical resonance of the piezoelectric element, nonlinear electrical properties of the coupled circuit, and the multiscale temporal structure as a result of the fast-slow decomposition of the FitzHugh-Nagumo formalism [22]. In a deterministic scenario, it has been known that such systems can exhibit limit cycle oscillations, bistability, and sensitive dependence on initial conditions, all applicable to the functional repertoire of neuromorphic devices that will be used in signal processing and pattern recognition [23]. Stochastic forcing, which is due to thermal fluctuations, device variability, or environmental noise, further enhances the dynamical landscape, introducing noise-driven transitions, stochastic resonance, and the destruction of the deterministic structure [24]. The study of these phenomena is therefore more than academic importance, but is also critical to the useful application of piezoelectric neurons within robust neuromorphic systems [25].

Ongoing research on piezoelectric neuron models has basically been qualitative, concerned with the behavior of the time-series in certain parameter ranges, or concerned with simple bifurcation diagrams showing the transitions between quiescent and oscillatory behavior [26,27]. These analysis give useful insights but are not exhaustive in describing the dynamical landscape as a whole. Especially, no systematic study of the geometry of the phase space such as the structures of the basins of attraction, the invariant manifolds, the return map or the stochastic recurrence quantification is performed. This restriction is noteworthy, because these geometric and statistical characteristics are essential to the sensitivity to initial conditions, multistability, long-term behavior, and sensitivity to noise. Thus, a detailed analysis is not provided, creating a clear gap in the research, and this gap is filled in this work. Another key factor is the fact that there was no strict stochastic study of the PNS. Although noise has been cited as a structurally important factor in the neural dynamics of both biological and artificial systems [28], its precise impact on the piezoelectric neuron model has not been quantified. Specifically, RQA which is an effective tool to define the predictability, determinism, and complexity of nonlinear time series under stochastic forcing [29], has not previously been applied to piezoelectric neuron systems. The mechanism of transition between deterministic and stochastic regimes and their implications on the recurrence structure, statistics of the trajectory, and geometry of the phase-space of the PNS is thus completely unexplored. In addition, a comparative statistical analysis of the fast activation variable $z_{1}$ and the slow recovery variable $z_{2}$ under stochastic forcing has not been provided and the differential sensitivity of the two subsystems to noise has not been systematically measured. Since the fast-slow form of the model is core to its excitability properties, such a gap forms a fundamental limitation of current knowledge on noise-driven dynamics in piezoelectric neuron systems [30].

In this study, the literature gap has been filled by formulating a systematic methodology to analyze a piezoelectric neuron model which is treated as a sensor inspired nonlinear dynamical system, both in deterministic and stochastic conditions. The model represents how mechanical perturbations convert to electrical responses that control nonlinear neuronal-like behavior. The proposed method combines the most recent developments in the nonlinear analysis techniques, such as the basin of attraction analysis, the phase space reconstruction, the Euler-Maruyama stochastic model and the RQA [31], to investigate the dynamics of a system under uncertainty. This gives a common view of the relationships between nonlinear dynamics and sensor applications and serves as a catalyst for a detailed study of deterministic and stochastic dynamics. The aim of this study is to systematically explore the same piezoelectric neuron model under deterministic and stochastic conditions. The overall research procedure starts with the formulation of the model, which translates mechanical perturbations into electrical responses controlling nonlinear neuronal-like dynamics. Basin of attraction mapping, sensitivity analysis with respect to initial conditions, phase space reconstruction and return map construction are used to analyze the behavior of the systems in the deterministic case to characterize limit cycles and nonlinear structures. The random perturbations are added with the Euler-Maruyama method in the stochastic situation and the dynamics of the ensemble are investigated by time-series analysis, phase portraits and stochastic RQA. Comparative analysis between deterministic and stochastic regimes and also between fast and slow variables ($z_{1}$ and $z_{2}$) is also carried out to pick up the multi-scale behavior. The framework highlights RQA features to characterize the impact of noise on the structure, predictability and dynamical robustness of the signal.

The paper is structured as follows: In Section 2, the mathematical modeling of the piezoelectric neuron system is explained. The perturbed analysis is explained in Section 3. Stochastic analysis is explained in Section 4. In Section 5, comparative trajectory and statistical analysis are explained. Then we have the closing remarks.

## 2. Mathematical Modeling of the Piezoelectric Neuron System

**Figure 1:**
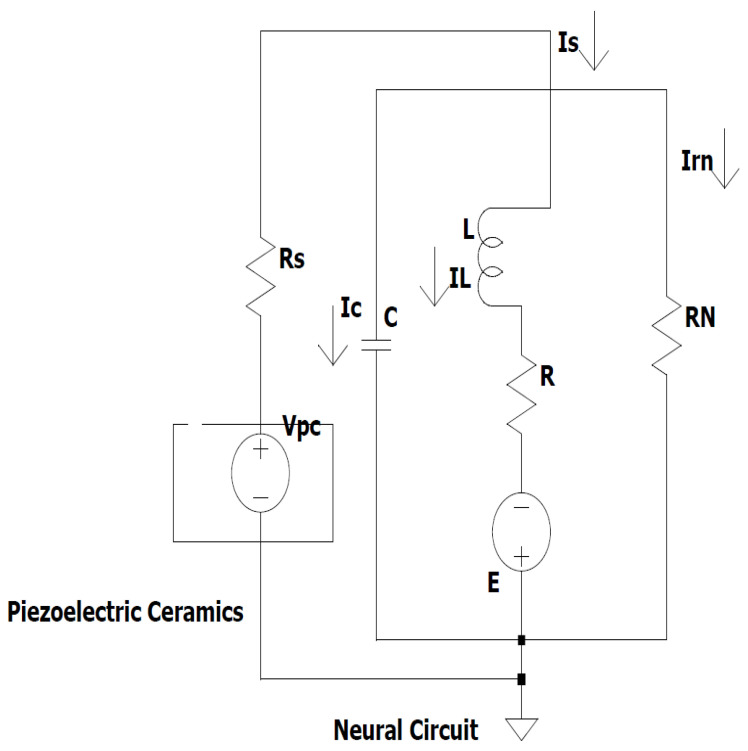
Piezoelectric neuron circuit model.

The practical circuit implementation of the proposed model of a piezoelectric neuron is shown in Figure 1. This circuit features a piezoelectric ceramic source that is connected to resistor-capacitor-inductor components, and a neural branch that mimics the electrical activity of a neuron. The energy transfer and oscillatory behavior of the circuit is controlled by the nonlinear resistance elements, the resistor $R_{s}$, the capacitor *C* and the inductor *L* which delivers the excitation voltage $V_{pc}$. These components interact with each other in ways that create complex non-linear dynamics and produce firing patterns similar to those of neurons. The feasibility of the proposed model is shown in such a circuit implementation and can be used as a basis for experimental implementation. The piezoelectric materials are under mechanical stress when an electrical charge is produced in the materials in proportion to the mechanical deformation involved, and these electrical signals can directly convert acoustic vibrations into electrical signals that can cause neural-like firing behavior. The functional architecture of the PNS resembles that of the human auditory transduction cascade. The piezoelectric element takes the role of the cochlear hair cell, which transforms mechanical deformation into a time-dependent electrical voltage which activates a nonlinear neural circuit.

At voltages higher than the excitation threshold of the circuit, action potential-like firing events are generated, which is similar to the spike encoding process of biological auditory neurons. With continued mechanical stimulation, the piezoelectric element experiences a continuous periodic strain, a temporal-dependent force that converts the electrical neural subsystem into periodic oscillatory, excitable, or chaotic firing states, with different stimulus amplification and frequency. This neural-mechanical-electrical-mechanical signal chain provides the PNS with threshold-dependent sensitivity and direct acoustic input, which is a physically realistic system in which neural response to mechanical stimuli can be studied and advanced neuromorphic architectures can be developed.

All things considered, PNS offer novel approaches to comprehending and interacting with the nervous system, marking a potential new area in neuroscience and bio-engineering.

The following equation explains how this deformation creates a potential difference between the terminals of the piezoelectric devices.

(1) $$\left\{\begin{array}{cc} V_{PC} & =\frac{F}{S}\frac{d_{33}}{\epsilon }h=Pgh,\\ P & =\frac{F}{S},\;g=\frac{d_{33}}{\epsilon },\;d_{33}=\frac{Q}{F}. \end{array}\right.$$

In this case, *F* stands for the external mechanical force, *S* for the cross-sectional area, ϵ for the dielectric constant, *h* for the thickness of the device, *Q* for the charge released and $d_{33}$ for the piezoelectric strain constant, which is related to the characteristics of the piezoelectric materials.

(2) $$\left\{\begin{array}{c} C\frac{dV_{C}}{dt}=\frac{V_{PC}-V_{C}}{R_{s}}-I_{L}-I_{RN},\\ L\frac{dI_{L}}{dt}=V_{C}-RI_{L}+E, \end{array}\right.$$

where *C* stands for capacitance and *L* for the coil self-inductance coefficient. $V_{PC}$ represents the voltage output across the piezoelectric ceramic, while $V_{C}$ represents the voltage across the capacitor. In the ion channel, *E* is the constant voltage source that simulates the reversal potential, whereas Rs and *R* are linear resistances. $I_{L}$ is the current flowing through the inductor, and the relationship between current and voltage across the nonlinear resistor $R_{N}$ is provided by:

(3) $$I_{RN}=-\frac{1}{\rho }\left(V-\frac{V^{3}}{3V_{0}^{2}}\right),$$

where $V_{0}$ and *V* represent the cutoff voltage and the voltage across the nonlinear resistor, respectively, and ρ is the normalization value. The dimensionless parameters in the following form can be added to the equation above to further simplify it:

$$z_{1}=\frac{V_{C}}{V_{0}},\;z_{2}=\frac{\rho I_{L}}{V_{0}},\;\tau =\frac{t}{\rho C},\;u_{PC}=\frac{V_{PC}}{V_{0}},\;\psi =\frac{\rho }{R_{s}},\;a=\frac{E}{V_{0}},\;\lambda =\frac{R}{\rho },\;\eta =\frac{\rho^{2}C}{L}.$$

The dimensionless dynamic equation of the PNS (2) can then be reduced to the following:

(4) $$\left\{\begin{array}{cc} {\dot{z}}_{1} & =z_{1}\left(1-\psi \right)-\frac{1}{3}z_{1}^{3}-z_{2}+\psi u_{PC},\\ {\dot{z}}_{2} & =\eta (z_{1}+a-\lambda x_{2}). \end{array}\right.$$

For the dimensionless time τ, the state variable is $z=[z_{1},z_{2}]$. When the external stimulation current $u_{PC}$ is varied, the systems dynamics may change to periodic, quasi-periodic or chaotic. In a physical sensing interpretation, a piezoelectric element is used to detect external mechanical stimuli, like sound waves, pressure changes or vibrations, and convert them into measurable electrical signals. Here, $z_{1}$ can be seen as the value of the electrical output from the piezoelectric sensor (such as a voltage signal), and $z_{2}$ as the internal state dynamics related to charge redistribution and/or relaxation effects in the material. The $u_{PC}$ is the input that enters the piezoelectric element, which is the mechanical stimulus given from the outside. However, in real-life piezoelectric sensing systems, the stimulus applied by an external mechanical source is usually a superposition of multiple frequency components, or mixed signals, of the source. These elements are the result of various sources of mechanical vibrations, resonances, and environmental disturbances that occur all at once on the piezoelectric element. Thus the actual excitation is a multi-frequency phenomenon. The present study deals with a simple baseline case involving a single-frequency input. This assumption leads to isolating and clarifying the basic nonlinear dynamic behavior of the proposed system without adding extra complexities due to the multi-frequency interactions. This type of simplification is often used in the initial theoretical investigations to provide a baseline response for the systems response. However, a mixed-frequency feature of the real piezoelectric signals is recognized as a significant physical effect, and the present theory could be extended to multi-frequency excitations to better reflect practical sensing environments. In the present study, the condition of single-frequency excitation was used as a baseline input condition so as to isolate and systemically analyze the basic nonlinear dynamics of the proposed piezoelectric neuron system. The use of forcing is simplified to allow the clear identification of attractor structures, sensitivity characteristics and stochastic effects without further additions of complexity from multi-frequency interactions. These two types of analysis, deterministic and stochastic, give a good idea of the intrinsic behavior of the system and offer a consistent framework for the nonlinear dynamical investigation.

In real piezoelectric sensing applications, however, the mechanical signals are typically multi-frequency or broadband. To partly explore this aspect, a small illustrative simulation using dual-frequency excitation was also explored. The results obtained show that the proposed system maintains the qualitative nonlinear behavior with stable attractor and structured oscillatory behavior even when subjected to mixed frequency forcing. The current setup can be straightforwardly extended to more realistic multilevel excitation in future research according to these observations. Thus, the model is a mathematical representation of the transformation of the various vibration patterns into different electrical responses in real piezoelectric sensing devices. The perturbation term is added to model environmental noise, the imperfection of the device and external perturbations, and hence realistic device behavior and transitions between various dynamical regimes typical in real-life sensing applications.

(5) $$\left\{\begin{array}{cc} {\dot{z}}_{1} & =z_{1}\left(1-\psi \right)-\frac{1}{3}z_{1}^{3}-z_{2}+A\cos\left(\Omega \tau \right),\\ {\dot{z}}_{2} & =\eta (z_{1}+a-\lambda z_{2}). \end{array}\right.$$

The parameter *A* represents the strength of the applied perturbation, which determines the strength of the energy injected into the system. Ω represents the angular frequency of excitation, which is the time scale by which the system is excited. Physically, this perturbation models either alternating mechanical loading (or) an AC electrical voltage on the piezoelectric material. Variations in *A* directly control the strengths of electromechanical coupling, which is capable of amplifying or damping oscillatory activity, but Ω controls resonance, synchronization, and transitions between periodic, quasi-periodic, and chaotic dynamical states.

A stochastic extension is added to the PNS to take into account the uncertainties of real-world neural environments.

Biological neurons are not deterministic at all: their behavior is persistently disturbed by thermal noise, ionic fluctuations, and changes in synaptic inputs. The inner ear hair cells in the sound context in particular convert mechanical vibrations caused by sound into electrical impulses under microscopic irregularity caused by ionic channel noise, change of temperature, and change of mechanical pressure. These sources of randomness imply that exactly the same acoustic inputs do not necessarily result in exactly the same neural responses.

In order to represent this biological fact, the PNS model is augmented with the addition of Gaussian white noise of zero mean, which is taken as a stochastic forcing term. This term adds unpredictable perturbations that change with time and makes the model reproduce the variability and uncertainty found in real auditory neural systems. Most importantly, although the system may start at the same initial conditions, stochastic forcing can cause a change between stable and unstable response regimes and a fundamentally different dynamical behavior of the system that cannot be seen in purely deterministic analysis. By including the stochastic term, the dynamical system (5) can be described as follows:

(6) $$\left\{\begin{array}{cc} dz_{1} & =\left[z_{1}\left(1-\psi \right)-\frac{1}{3}z_{1}^{3}-z_{2}+A\cos\left(\Omega \tau \right)\right]\;d\tau +\sigma_{1}\;dW_{1}\left(\tau \right),\\ dz_{2} & =\eta (z_{1}+a-\lambda z_{2})\;d\tau . \end{array}\right.$$

The stochastic analysis is performed with the addition of a Gaussian white noise to the system to account for random external perturbations. It is assumed that the noise has zero mean and intensity σ which determines the strength of stochastic excitation. The noise is referred to as delta-correlated in time (no correlation time) which is a standard white noise process. To systematically explore the effects of stochasticity on the system dynamics, various values of the noise strength σ are explored, allowing them to study weak to strong noise regimes. Euler-Maruyama method is used to numerically solve the stochastic differential equations. In the proposed stochastic formulation, Gaussian white noise is added in the fast state variable $z_{1}$ only. The selection is guided by the physical and dynamical dynamic of the system variables. In particular, $z_{1}$ is related to the fast activation/output dynamics, which are directly influenced by external fluctuations, environmental disturbances, and measurement uncertainties. A slower recovery or adaptation process, with a longer time scale, is described by $z_{2}$ and is generally less responsive to rapid stochastic perturbations. The simplifying assumption is therefore to only stochastically force $z_{1}$ to reflect the dominant source of variability in the fast response, but to keep the model simple and retain the stability of the slow subsystem. This modeling assumption is similar to what can be found in neural or nonlinear oscillator systems where noise is usually added to the fast dynamics to account for noise from outside the system as well as for internal fluctuations.

The parameter values and initial conditions used throughout the numerical simulations are described in the following. All simulations share the fixed baseline parameters ψ=0.45, η=0.12, a=0.4, and λ=0.18, which remain constant in all experimental configurations. The sets of three parameters differ exclusively in amplitude *A* and angular frequency Ω, which are systematically varied to investigate different dynamical regimes. Specifically, Set 1 uses A=0.75 and Ω=1.2, representing a low-amplitude low-frequency excitation regime. Set 2 employs A=0.85 and Ω=1.6, corresponding to a moderate excitation regime, while Set 3 applies A=1.3 and Ω=1.9, representing a high-amplitude, high-frequency forcing regime.

Each parameter set is simulated under three distinct initial conditions to examine the sensitivity of the system to its starting state. The initial Condition 1 (IC1) places the system near the origin at $\left(z_{1},z_{2}\right)=\left(0.05,-0.03\right)$, representing a starting state near-equilibrium. The initial Condition 2 (IC2) initializes the system at $\left(z_{1},z_{2}\right)=\left(0.8,0.2\right)$, corresponding to a moderately displaced state, while the initial Condition 3 (IC3) places the system far from equilibrium at $\left(z_{1},z_{2}\right)=\left(-1.2,1.6\right)$, representing a strongly perturbed initial state.

The full combination of three parameter sets and three initial conditions yields nine simulation configurations in total, enabling a systematic and comprehensive exploration of how perturbation strength, excitation frequency, and initial state jointly govern the dynamical behavior of the piezoelectric neuron system. All simulations were carried out with Python (version 3.13.2) and the scientific libraries NumPy, SciPy and Matplotlib. The installed library versions are NumPy 2.2.4, SciPy 1.15.2, and Matplotlib 3.10.1. The system also uses the built-in Python sys module. The deterministic system was solved numerically with a SciPy function, odeint, which is based upon the LSODA algorithm with automatic step-size control. The simulations were performed on the time interval τ∈[0,300], with 20,000 equally spaced time points, thus a time step of Δτ=0.015. The default tolerance parameters of the solver were applied. The numerical integration of the stochastic system was carried out with the Euler-Maruyama method. The simulations were carried out in the range τ∈[0,100] with 5000 time steps, and thus Δτ=0.02. The stochastic term was expressed as the Gaussian white noise σ=0.1 acting on the first state variable. A fixed random seed The notation np.random.seed(42) refers to the fixed random seed used to ensure reproducibility of stochastic simulations and is not a reference citation.(np.random.seed(42)) was employed to ensure the same results of the stochastic simulations.

## 3. Perturbed Dynamical Analysis

In this part, the perturbed dynamics of the system are discussed to investigate the effects of important parameters on the stability and firing of the piezoelectric neuron system. The system also has a strong deterministic structure, which allows systematically varying the parameters to obtain a complete characterization of the neural responses to mechanical stimuli to give a rigorous basis on the fundamental mechanisms that control complex auditory neural dynamics. Quasi-periodic behavior many nonlinear dynamical systems, among which are the PNS, are dynamical systems where the trajectories are on a multi-dimensional torus in phase space instead of being closed periodic orbits. Quasi-periodicity is a motion induced by interaction of two or more incommensurate frequencies and is structured and limited, but never repeats precisely. This dynamical regime has a middle ground between chaotic and regular periodicity: unlike chaotic orbits, quasi-periodic ones are not sensitive, but unlike purely periodic orbits, they occupy some areas of phase space and do not recur. On an auditory background, quasi-periodicity can occur naturally when two or more incommensurate sound frequencies simultaneously stimulate the neural circuit to generate complex and yet well-organized firing patterns to indicate the spectral richness of the acoustic input. Nonlinear circuit dynamics and quasi-periodic external forcing may also coexist, further causing transitions to chaotic regimes, and hence quasi-periodicity is a dynamically important state which marks the transition between predictable and unpredictable neural responses in the PNS.

**Figure 2:**
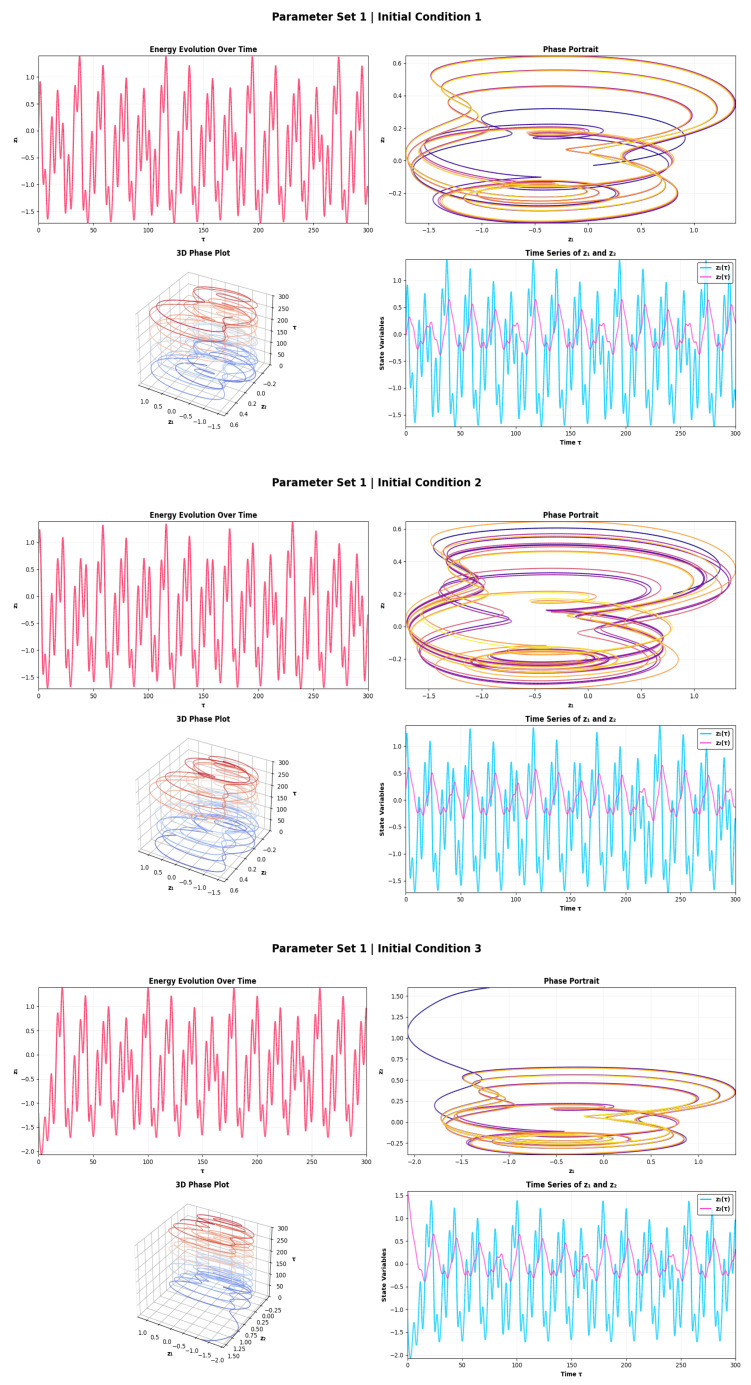
Energy evolution, phase portrait, 3D attractor, and time series plots for System (5) using parameter set 1.

**Figure 3:**
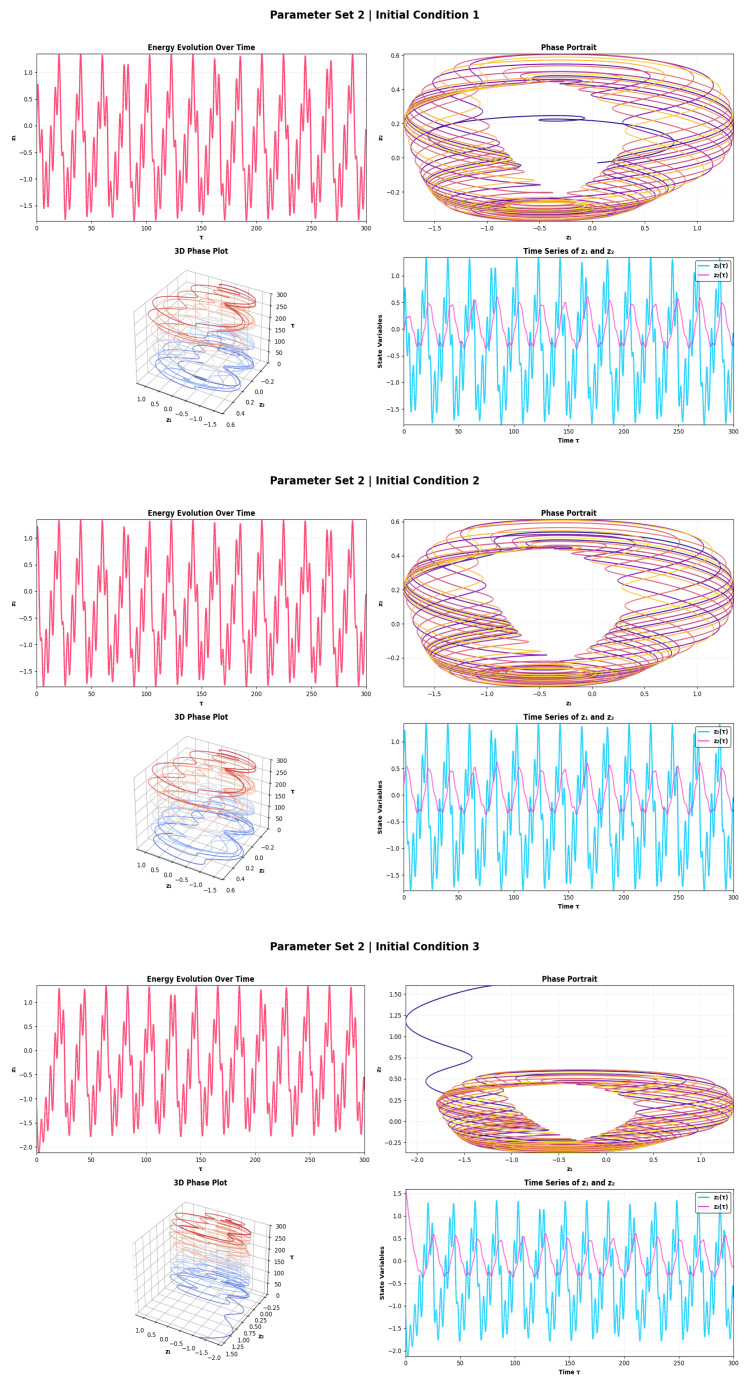
Energy evolution, phase portrait, 3D attractor, and time series plots for System (5) using parameter set 2.

The dynamical behavior of the PNS for Parameter Set 1 over three initial conditions is presented in Figure 2. The amplitude of the energy evolution is seen to undergo periodic oscillations with near-constant amplitude (peak-to-peak ≈3.0–3.2 units, coefficient of variation <2%), which is indicative of convergence to a stable limit cycle with dominant frequency $f_{1}\approx 0.08$–0.10 Hz. This steady, low-energy firing mode is signifying a tonic firing mode, which implies that there will be repeatable and reliable signal detection with restoring forces in the system exceeding the nonlinear excitation. The phase portraits contain concentric closed curves in the annulus (phase space area ≈ 4.5–5.0 square units) and the 3D plots confirm the presence of a bounded toroidal attractor. The presence of a fast mode ($f_{1}$) in $z_{1}$ with the peak amplitude of ≈1.2–1.5 units and a slow mode ($f_{2}\approx 0.02$–0.03 Hz) in $z_{2}$ with the peak amplitude of ≈0.3–0.5 units confirms quasi-periodic dynamics suitable for frequency-sensitive sensing. The RR (≳0.85) is very high, and the convergence of all orbits is within ≲50 time units, even for different initial conditions. The strong attractor contraction under noisy excitation yields bounded energy fluctuations, without destroying the limit cycle, and is the most stable and reproducible regime under noise. Figure 3 illustrates the dynamics for Parameter Set 2 and shows how the forcing amplitude influences the dynamics. As a result, the firing regime evolves into a modulated firing regime with an energy amplitude increasing to ≈4.5–5.0 units, corresponding to the coefficient of variation of ≈8–12% which is a ∼50% increase over the value from Parameter Set 1. This modulation occurs as a result of beating between incommensurate frequencies $f_{1}$ and $f_{2}$ and creates slow amplitude envelopes, which provides greater temporal encoding than tonic firing. The phase portraits as well as the RQA determinism DET >0.90 and an area of phase space ≈7.0–8.5 sq. units of the torus with a hollow core of diameter ≈ 0.6–0.8 units indicate structured non-chaotic dynamics. Under IC 3, the transient settling goes on for about 80–100 time units. The quasi-periodic torus is more sensitive than the limit cycle of Parameter Set 1: noise of low intensity induces phase slips between frequency modes of the torus, leading to the release of energy bursts which can be used for sub-threshold signal detection through the stochastic resonance effect. Parameter Set 2 is then the most noise sensitive and is most appropriate for weak-signal detection applications.

**Figure 4:**
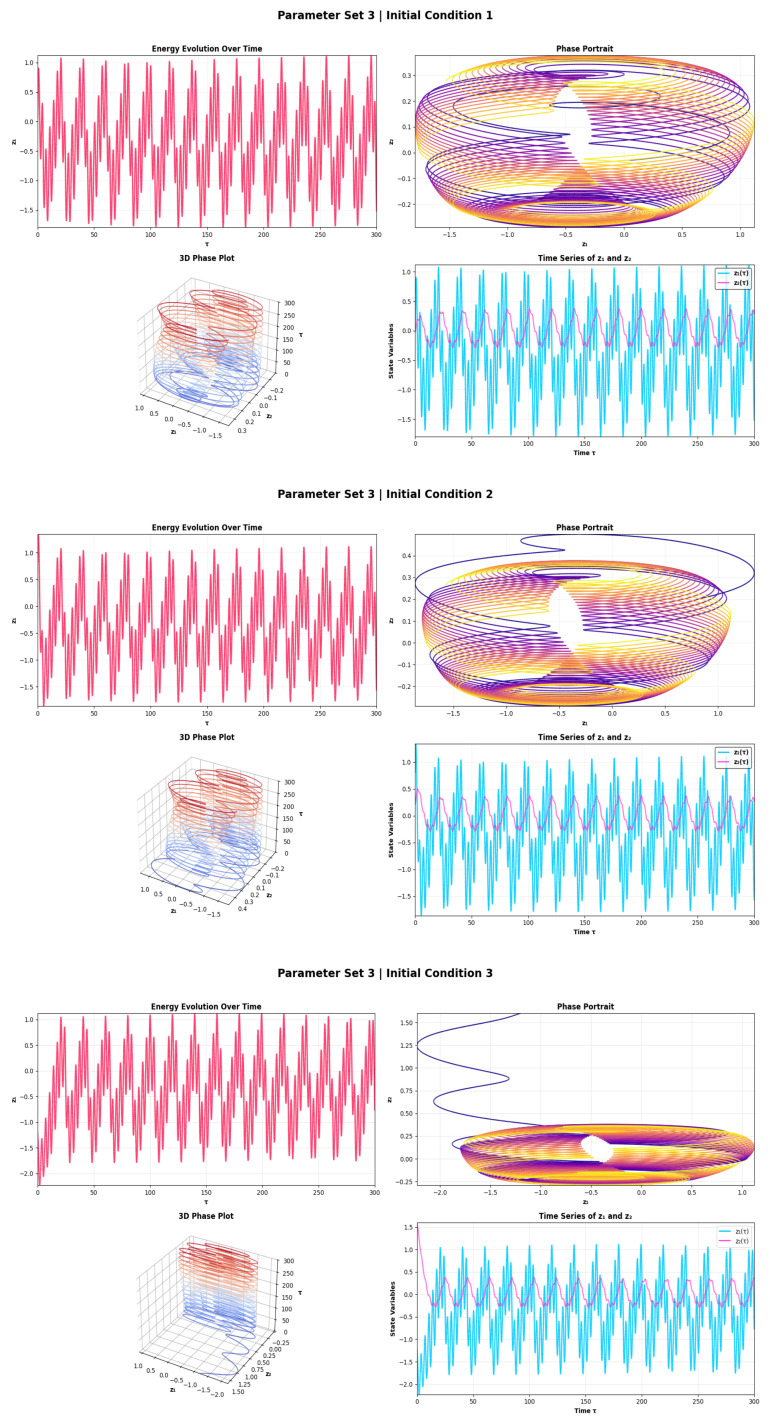
Energy evolution, phase portrait, 3D attractor, and time series plots for System (5) using parameter set 3.

For Parameter Set 3 the most effective forcing is shown in Figure 4. The firing regime for the energy amplitude is modulated and is found to be at high energy with a coefficient of variation of ≈14–18% and the phase space area has expanded to ≈9.5–11.0 units with a hollow core diameter of ≈0.9–1.1 units, which corresponds to a high-energy modulated firing regime near but not in chaotic breakdown. All three parameter sets display a progressive thickening of the toroidal attractor, which represents a direct signature in the energy domain of the transition between tonic firing and modulated firing, and can be used as a quantitative discriminator of the operating mode. The qualitative measure of recurrence entropy is maximum, DET ≳0.88 here, which indicates maximum dynamical richness but not determinism. The amplitude of oscillations is highest ($z_{1}$ ≈ 1.8–2.2 units, $z_{2}$ ≈ 0.6–0.8 units) and the IC 3 has the longest transient (≈120–150 time units) in line with the higher sensitivity in this regime. The simultaneous appearance of $z_{1}$ and $z_{2}$ input variations can be realized by the fast-slow coexistence of $z_{1}$ and $z_{2}$ which is a natural multi-time-scale processing mechanism. For moderate noise, the effect on energy statistics is relatively small for the systems in Parameter Sets 1 and 2 in the presence of noise, due to the systems intrinsic variability already dominating the energy response envelope for this situation.

A quasi-periodic behavior is observed, which improves the ability of the system to sense than simple periodic oscillations. The presence of several incommensurate frequencies allows the system response to be utilized in both amplitude modulation and frequency modulation. The multi-frequency structure allows to better represent the signal and to better capture the complex external stimuli. Moreover, due to the co-existence of fast and slow time scales, the system can detect different inputs variations simultaneously, which is suitable for multi-scale sensing applications.

### 3.1. Basin of Attraction

Long-term behavior of a dynamical system cannot be completely determined by the governing equations, but also heavily relies on the initial conditions. Here, the basin of attraction refers to all those initial states that tend to approach a particular attractor. These basins are geometrically structured, and the geometric information is useful in terms of multistability, robustness and sensitivity of the system in the phase space that is especially useful in terms of determining response reliability in nonlinear sensor-like systems. The basin of attraction maps are created by numerically sampling a uniform grid of initial conditions in the $z_{1}$–$z_{2}$ phase space. A grid size of N=300 is employed for each basin plot, which is equal to 300×300 initial conditions pairs. To simulate each of the trajectories, a total time T=100 is taken with a time step dt=0.05. The attractor behavior is classified from the final state of the trajectory obtained numerically for each initial condition using the system to integrate it. The value of $z_{1}$ at the final time is used as the classification indicator. This high sampling rate provides a good resolution of basin boundaries and a reliable identification of attraction regions in the phase space.

**Figure 5:**
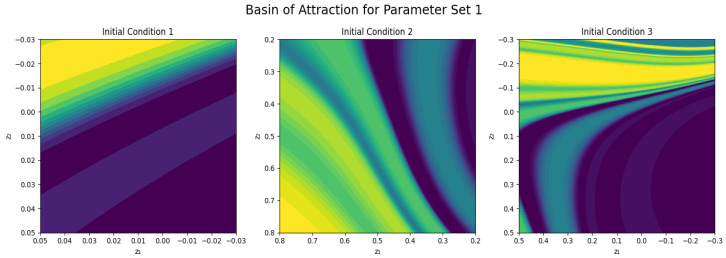
Basins of attraction for parameter set 1. Colors indicate which attractor each initial condition converges to, revealing the system sensitivity to initial states and the structure of its dynamical landscape.

We present the corresponding basin structure for the PNS with different neighborhoods of initial condition with the help of Figure 5 for Parameter Set 1. Regions are yellow when the trajectory flows towards the positive attractor, and dark purple when the trajectory flows towards the negative attractor. The intermediate colors indicate areas of basin boundaries, in which the system is sensitive to small changes in the initial conditions. The smooth and well-separated basin regions suggest a relatively simple and predictable dynamical regime for IC1 because small perturbations near the basins will have a predictable response. However, IC2 displays curved and distorted basin boundaries, interpreted as being influenced by nonlinear piezoelectric forcing and intrinsic neural activity, with small changes in initial conditions resulting in qualitatively different long-term dynamics. The basin structure of IC3 is highly complex and interwoven, which implies that there is high multistability, with several attractors existing and initial conditions close to the attractor that evolve to a variety of dynamical states.

### 3.2. Sensitivity Analysis

**Figure 6:**
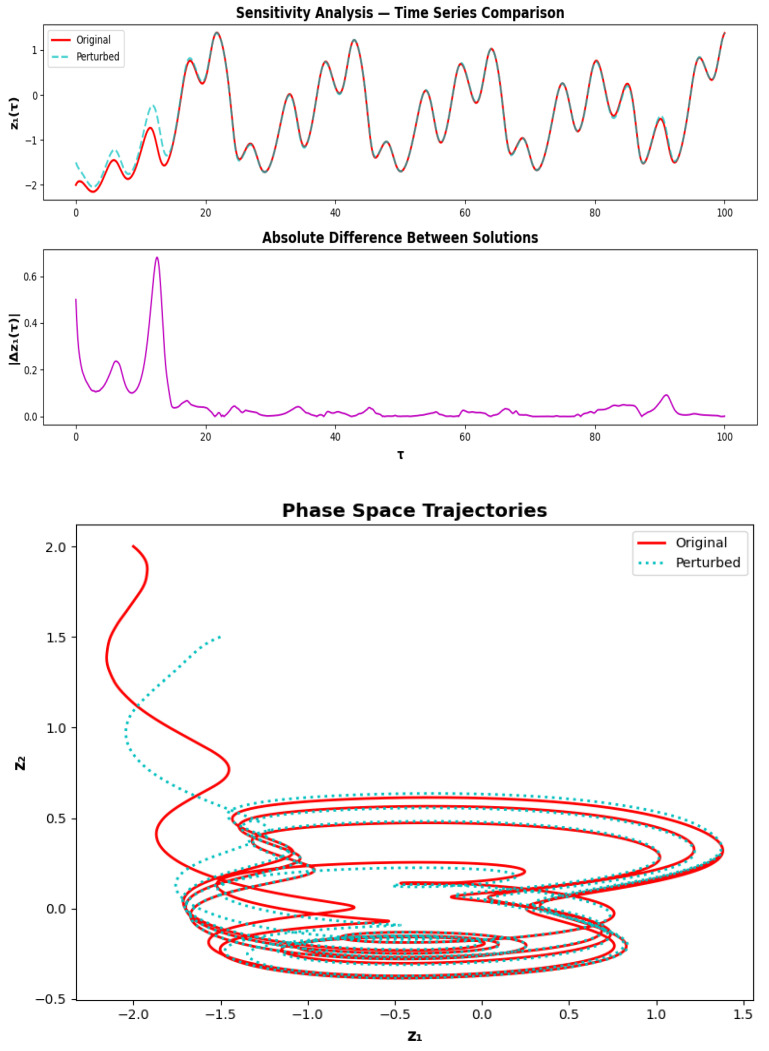
Perturbed case. (**Top**) Time series of $z_{1}\left(\tau \right)$ for both original and slightly perturbed initial conditions. (**Middle**) Absolute difference $|\Delta z_{1}\left(\tau \right)|$ between the two trajectories. (**Bottom**) Phase space plot showing trajectories in the $z_{1}$–$z_{2}$ plane.

Sensitivity analysis quantifies how infinitesimal perturbations to the initial conditions of a dynamical system propagate and evolve over time, providing a rigorous measure of the systems predictability and structural stability. For System (5), a small perturbation δ is applied to the initial state of the activation variable $z_{1}$, and the subsequent divergence between the original and perturbed trajectories is tracked through a time-series comparison, absolute difference evolution, and phase space geometry. Figure 6 shows a non-trivial and physically meaningful sensitivity structure. The time-series comparison in the upper panel reveals that the initial transients of the two trajectories are sensitive to initial conditions, typical of nonlinear excitable dynamics. This is in contrast to a divergent system where the absolute difference $|\Delta z_{1}\left(\tau \right)|$ (middle panel) grows at first, peaks around τ≈10–15 and then decreases to a small residual value. This behavior shows that the trajectories end up converging back toward the same underlying attractor. The separation between perturbed trajectories can shed light on the quantitative measure of the transient divergence and reconvergence. The decay of $|\Delta z_{1}\left(\tau \right)|$ is roughly exponential, so that the system is quickly stabilized and highly resistant to initial conditions. The presence of a stable attracting set with a finite basin of attraction is confirmed by this transient behavior followed by an asymptotic decay, even if it is short-time sensitive.

The phase space plot below confirms this understanding. The original and perturbed trajectories take different transient paths in the $z_{1}$–$z_{2}$ phase plane and the perturbed trajectory makes a larger initial excursion before it is attracted to the attractor. Eventually both paths lead to the same finite geometric object, a stable family of quasi-periodic orbits. This convergence indicates that the attractor geometry is robust against initial conditions variations and to small perturbations. In general, the system is both very sensitive when it comes to transient evolution (which makes it possible to distinguish between initial states) and also very good at converging on a stable attractor. For piezoelectric neuromorphic systems to have a strong coding of signals, this balance among sensitivity and stability is necessary in realistic operating conditions.

### 3.3. Return Map

**Figure 7:**
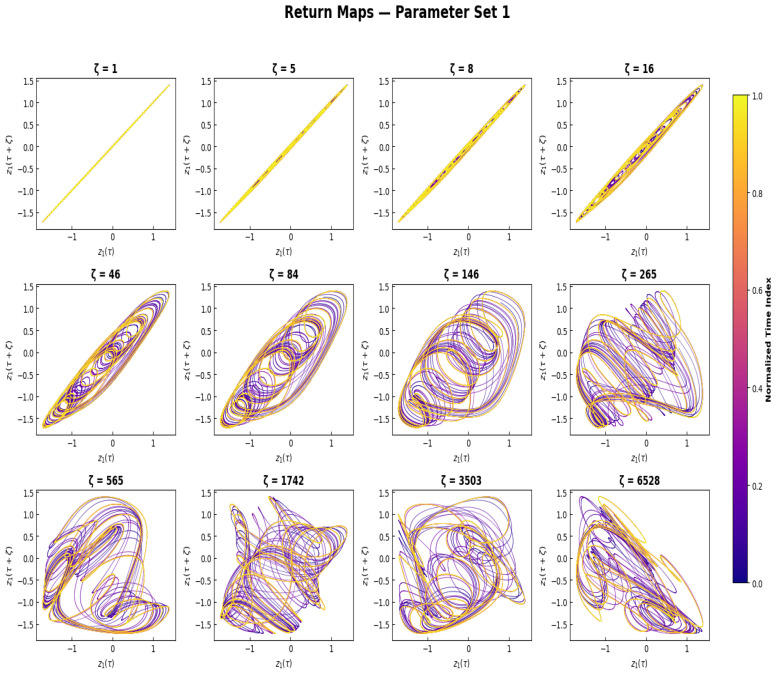
As delay ζ increases, the delay return maps display $z_{1}\left(\tau +\zeta \right)$ vs. $z_{1}\left(\tau \right)$. Chaotic dynamics is characterized by a lack of correlation at greater delays and a nonlinear perturbed structure at intermediate delays, which are reflected in the evolving geometric complexity.

A delay return map recreates the time structure of a dynamical system by plotting an observable $z_{1}\left(\tau \right)$ against its delayed counterpart $z_{1}\left(\tau +\zeta \right)$, and giving a low-dimensional geometric projection of the underlying attractor. This representation makes it possible to directly to visually differentiate dynamical regimes a diagonal line represents short-term predictability, smooth closed curves represent quasi-periodic dynamics, and folded or diffuse structures represent chaotic or high-dimensional dynamics. The delay return maps are plotted for twelve growing delay values, with the colour map normalized and used to show the time evolution of the map structure, as seen in Figure 7. The return maps for small delay values collapse onto an almost perfect diagonal line, meaning that there is strong short-term autocorrelation and smooth evolution of the trajectories along the attractor. When the delay is increased to intermediate values, the diagonal structure slowly evolves into smooth elliptical and looped patterns, that correspond to the underlying quasi-periodic geometry of the attractor when the delay is greater than the correlation time of the system. The return maps have more complicated folded and interwoven structures at larger delay values, with a wider area of the phase space being occupied, suggesting a decrease in temporal correlation and an increase in geometric complexity in quasi-periodic motion on a toroidal attractor. Importantly, even at the largest delays considered, the return maps do not show sharp discontinuities or fully homogeneous space-filling patterns which are characteristic of fully chaotic dynamics. This is evidence that the PNS does not become chaotic, but rather exhibits structure over a number of time scales. The observed evolution of the return map geometry from strong diagonal coherence to complex but structured pattern suggests overall a multiscale temporal organization, from short term predictability to long term decorrelation. Such behavior is consistent with the requirements of multiscale signal processing for biological auditory systems and thus the PNS could provide a potential platform for neuromorphic auditory signal encoding.

### 3.4. Recurrence Plots

A recurrence plot (RP) is a graph used to visualize the instances of a dynamical system revisiting roughly the same part of the phase space and to build a symmetric binary matrix in which a recurrence point is indicated when two states are within some specified distance. The dynamical aspect of the system is directly reflected in the geometry structures that are incorporated in the RP: diagonal lines imply periodic or quasi-periodic reoccurrence, isolated points imply chaotic dynamics, and horizontal or vertical lines imply laminar phases. When applied to the PNS, recurrence analysis offers a potent method to identify dynamical changes of the system, predictability metrics, and describe the structural response of the system to harmonic perturbation in each of the three set of parameters.

For the RP, the phase space was created from the simulated trajectory $\left(z_{1},z_{2}\right)$ and therefore in this case the embedding dimension m=2 is equal to the system dimension. The time delay is taken as one integration time step of the numerical solution. The cosine distance is used to calculate the recurrence matrix and the threshold for recurrence is set to ε=0.05, which is a good compromise between sparsity and structure on the recurrence plot. The type of normal is thus explicit and cosine-based. The recurrence quantification measures are calculated as follows: the recurrence rate is given by the density of recurrence points in the matrix, determinism is given by diagonal line structures with a minimum diagonal length, $l_{\min}=2$, laminarity is calculated from vertical structures with a minimum length, $v_{\min}=2$ and entropy is calculated from the probability distribution of the length of diagonal lines. These parameter values guarantee that the system dynamics, measured in terms of recurrence structure, predictability, and laminar behavior is consistent and reproducible.

**Figure 8:**
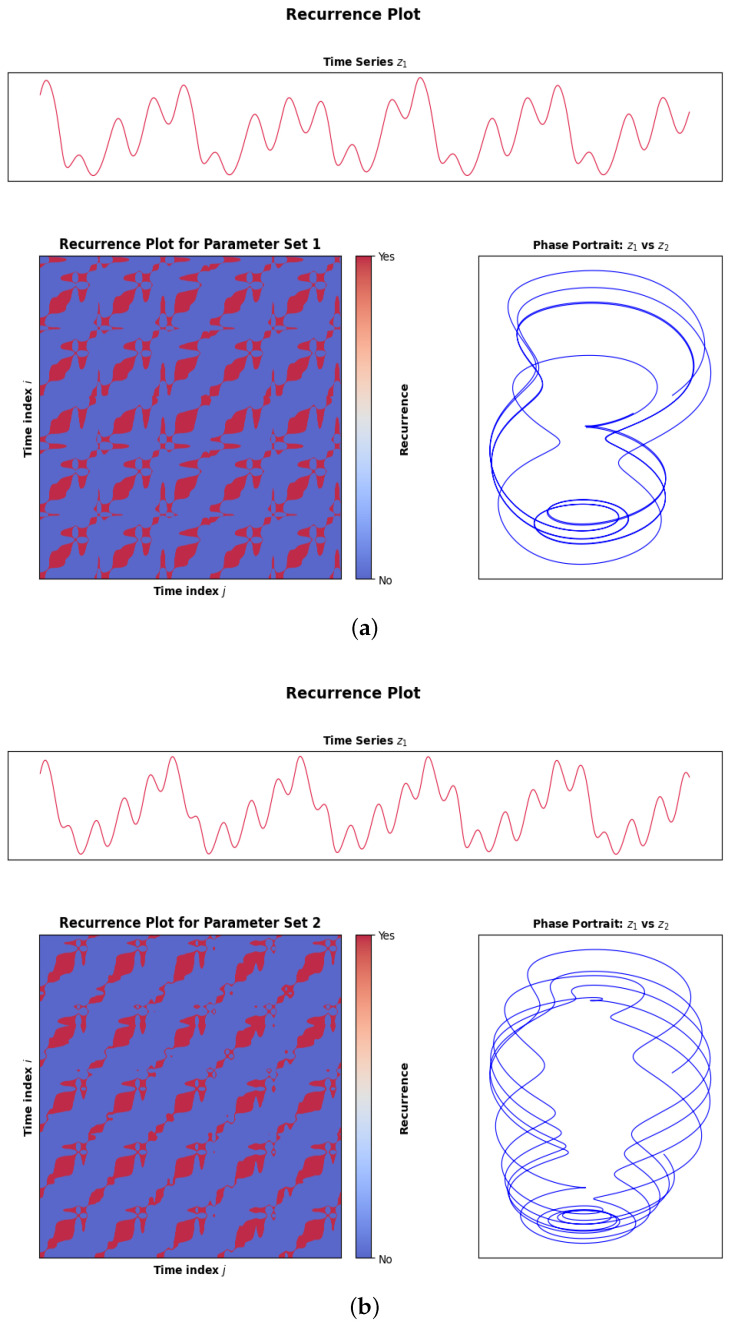

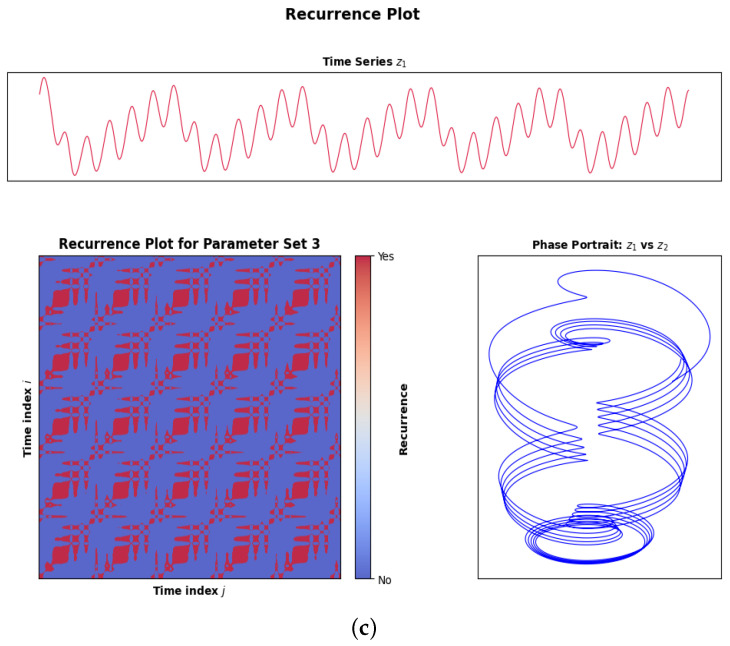
Recurrence analysis of system dynamics, (**a**–**c**) show results for different parameter values, including time series, recurrence plots, and the corresponding phase portraits.

In the time series shown in Figure 8a, irregular oscillations are observed on a small scale, while in the phase portrait (Figure 8b), a well-defined toroidal attractor is clearly visible. The recurrence plot of the system shows rich diagonal line structures in an organized non-random background, demonstrating the existence of structured quasi periodic recurrence. Recurrence points are not uniformly distributed, so this system is not fully chaotic, and perfectly continuous diagonal lines are absent, meaning the system is not perfectly periodic and is instead quasi-periodic. The recurrence structure shown in Figure 8b is more spatially homogeneous, and the diagonal structure is more coherent and prominent, suggesting that there is more quasi-periodic organization. This is confirmed by the phase portrait, which displays a more regular attractor geometry and a smaller transverse spreading rate, indicating a higher level of temporal regularity and lower dynamical complexity in the system when it is forced as in this case. The greater the structural complexity, the more the recurrence plot in Figure 8c is packed with structures and has sparks of structures occurring at intervals. The attractor is bounded under stronger forcing; the phase portrait shows more geometric complexity in the attractor, indicating it is near a transition regime or bifurcation boundary. Most important, no point scattering is observed, suggesting that full development of chaos has not yet occurred. The overall pattern of recurrence plots shows an evolutionary pattern of dynamics in the system, from quasi-periodic structures to higher coherence and more complex structures, indicating the multi-scale dynamics in PNS that can not be detected from time series or phase space analysis.

## 4. Stochastic Dynamical Analysis

**Figure 9:**
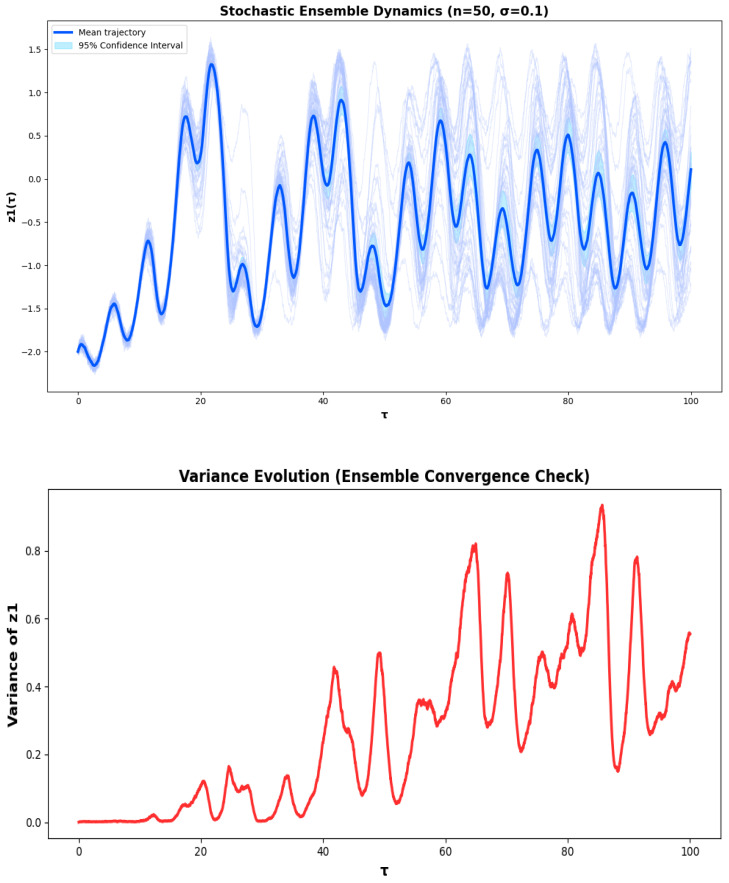

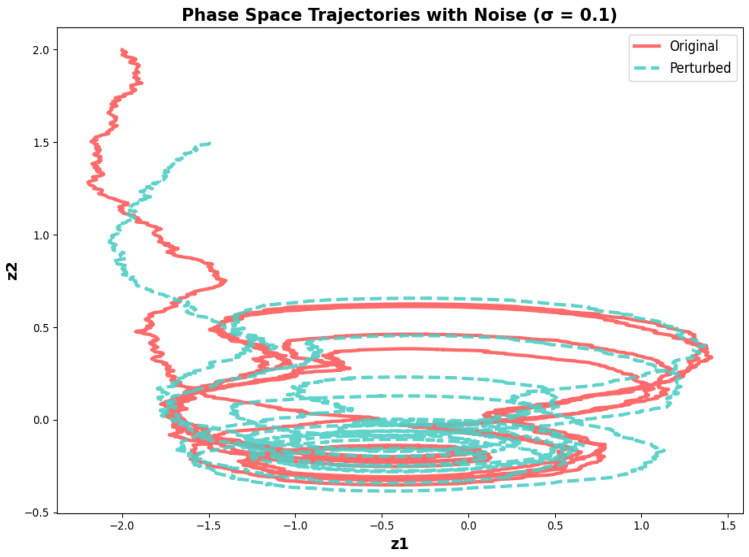
Ensemble stochastic dynamics of $z_{1}\left(\tau \right)$ showing individual realizations, mean response, and 95% confidence interval, together with phase space trajectories illustrating the system evolution and variability under stochastic excitation.

Although deterministic analysis exposes the inherent geometric arrangement of the PNS, reality piezoelectric and biological neural systems function under the conditions of sustained random fluctuation due to thermal noise, ionic variability, and mechanical irregularities. In order to model this physical reality, additive Gaussian white noise is added to System (6) to model the trajectory by an ensemble of stochastic realizations. This analysis measures the change in stability, oscillatory structure, and the geometry of the phase space of the PNS to random variation and whether the underlying attractor is robust when subjected to noise. For an ensemble of numerical analysis, the Euler-Maruyama method is used on the system of the stochastic behavior investigated in Figure 9. To provide statistical reliability and minimize sampling bias 50 independent stochastic realizations are generated. The dynamical behavior of the ensemble trajectories of the individual $z_{1}\left(\tau \right)$ is seen to be very different, and the differences can be attributed to the stochastic perturbations. The ensemble mean response, however, shows a stable well defined trend indicating the average dynamical response of the ensemble. The dispersion of the stochastic trajectories around the mean solution is clearly identified in the ensemble statistics and a 95% confidence interval is calculated from this ensemble. This gives a quantitative figure of the variation of systems in noised conditions. Also, the time variation of variance is also examined to determine the convergence of the ensemble statistics. The bounded behavior of variance shows that the stochastic realizations are close together statistically, validating the selection of the number of ensemble of realizations used to produce reliable characterization of the dynamics of the system. In general, the results confirm that each trajectory shows stochastic variability, but the system has a stable statistical structure in the ensemble, thus corroborating the validity of the numerical results. The phase space plot also shows that both deterministic and stochastic trajectories converge to the same attractor in the $z_{1}$–$z_{2}$ plane. The structure of the attractors is unaffected by the presence of noise, only the geometry of each of them is perturbed locally. This clearly shows that only local variations in the trajectory are stochastic, while the system structure is determined by deterministic dynamics, thus supporting the robustness of the attractor. In order to more deeply explore the stochastic dynamics of the proposed system, coherence resonance is studied in the presence of external noise. It is found that the oscillatory responses are more coherent and regular when a suitable noise intensity is added to the system, and that the firing behavior of a group of neurons is more coherent. The phenomenon suggests that the noise is not to be considered destructive, but rather has a constructive role in the piezoelectric neural circuit, bringing improvements in the temporal ordering of the system dynamics. The results obtained coincide with the presence of coherence resonance and show the sensitivity of the proposed neuron model to stochastic perturbations.

## 5. Comparative Trajectory and Statistical Analysis

### 5.1. Phase Space Comparison

**Figure 10:**
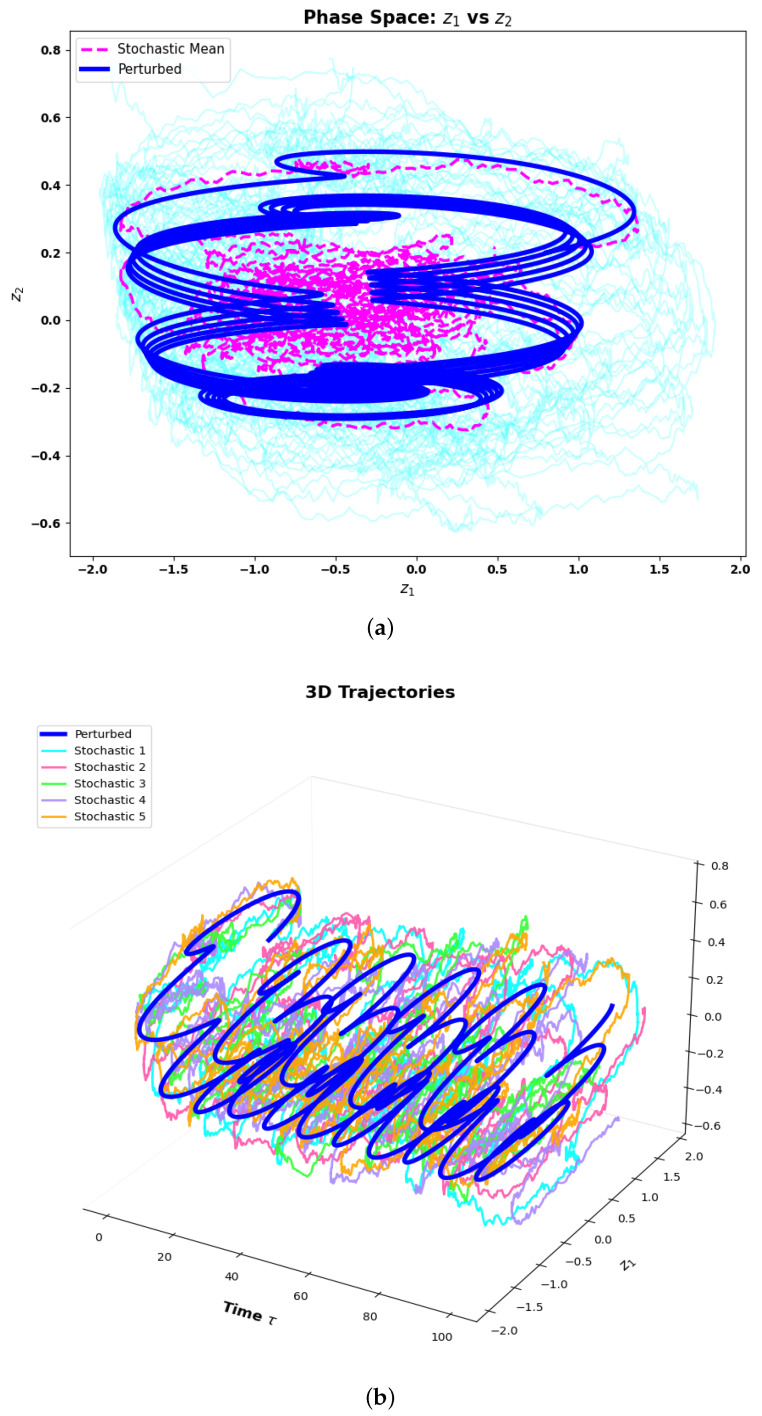
Analyzing recurrence plots stochastically: (**a**) Phase space comparison: In the $z_{1}$–$z_{2}$ phase space, perturbed and stochastic trajectories are compared. (**b**) 3D trajectories: $\left(z_{1},z_{2},\tau \right)$: A visual representation of the systems temporal evolution, showing both stochastic and perturbed trajectories over time.

In order to study the deterministic perturbation and stochastic fluctuations affecting the dynamical behavior of the PNS, a direct comparative study is conducted between the perturbed deterministic trajectory and an ensemble of the stochastic realizations of the two-dimensional phase space representation and the three-dimensional phase space representation. The phase space of the perturbed point of the deterministic trajectory and stochastic ensemble are shown in Figure 10a. The perturbed deterministic orbit has a regular, well structured orbit in the region of the phase plane and the orbit is always the same, showing the stability and repeatability of the deterministic attractor. The geometric coherence and continuity of this orbit is a strong confirmation of the quasi-periodic nature of the underlying dynamics; that is, the motion is smooth and wraps around on a toroidal attractor surface while being highly long-term predictable.

The phase plane of the stochastic realizations, on the other hand, shows much more dispersed spatial distribution for the phase plane. The distance from the core of the attractor is an example of a distance leading to a nonlinear dependence of individual stochastic trajectories from the deterministic one. The spatial spreading is found to be due to the cumulative effect of stochastic forcing that causes fluctuations in the trajectories from the deterministic flow and adds irreducible variability in the phase space structure. In spite of this diffusion, the mean trajectory of the ensemble maintains most of the global geometric structure of the deterministic trajectory, suggesting that the attractor still has an organizing effect even in the face of the noise. The three dimensional trajectory plot Figure 10b further supports these observations. The deterministic trajectory is organized in a coherent helical structure in the extended phase space, while the stochastic trajectories are organized around a diffuse cloud that fluctuates in response to persistent noise. The deterministic backbone is clearly visible inside this stochastic envelope, which implies that the system’s backbone retains its dynamical identity despite its random perturbations. In parallel, the stochastic spread captures the type of variations common in neural systems under realistic conditions of noise. In conclusion, this structural coherence-stochastic variability relationship demonstrates the robustness and adaptability of the proposed piezoelectric neuron system for reliable neuromorphic signal processing.

### 5.2. Trajectory Comparison of $z_{1}$ and $z_{2}$

**Figure 11:**
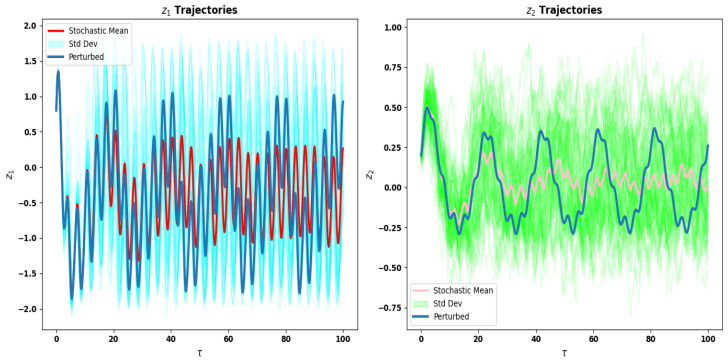
Comparison of perturbed and stochastic trajectories of $z_{1}$ and $z_{2}$.

A direct comparison between the deterministic perturbed trajectories and the stochastic ensemble is given in Figure 11 for both state variables, giving a quantitative visualization of the influence of noise on the fast activation variable $z_{1}$ and the slow recovery variable $z_{2}$ throughout the simulation time interval.

The stochastic ensemble shows larger variability band around the deterministic perturbed trajectory for $z_{1}$ and significant and frequent deviations from the deterministic perturbed trajectory. This is suggestive of a high sensitivity to stochastic perturbations for $z_{1}$ which is the fast activation variable and hence subject to rapid nonlinear dynamics. The stochastic mean is still able to follow the deterministic trajectory in general, but there is a considerable spread due to the high instantaneous uncertainty in the system response, yet retaining the global oscillatory structure.

The behavior of $z_{2}$ on the other hand, is clearly less sensitive to noise. The members of the stochastic ensemble stay close to the deterministic perturbed trajectory during the simulation, and the spread between the stochastic realizations and the deterministic trajectory is relatively small. This is due to the slow timescale of $z_{2}$ which filters out high-frequency stochastic variations and prevents fast responses to noise. This leads to high structural coherence and robustness of the slow subsystem, as the mean of the stochastic solution is close to the deterministic solution.

In general, the overall difference in the noise sensitivity of $z_{1}$ and $z_{2}$ showcases the intrinsic fast–slow decomposition of the PNS. This multiscale behavior means that the fast variable controls the fast and noisy responses, while the slow variable controls the backbone of the system dynamics that keeps the dynamics stable to stochastic forces, important for reliable signal processing in neuromorphic applications.

### 5.3. Statistical Distribution

**Figure 12:**
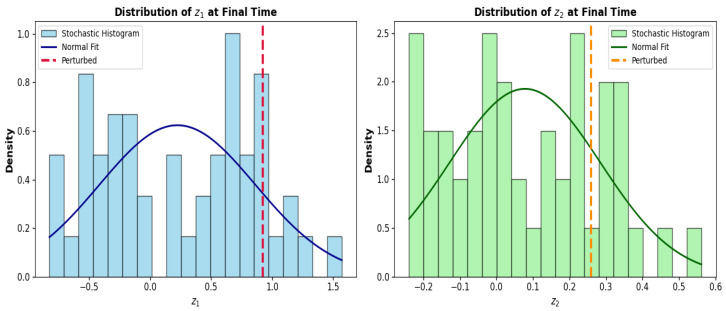
Probability density distribution of $z_{1}$ (**left**) and $z_{2}$ (**right**) at the final time step. Normal fits are shown in blue and green, and perturbed final values are marked with vertical dashed lines.

The final time distributions of $z_{1}$ and $z_{2}$ give a statistical characterization of the effect of the stochastic forcing on the system compared to the deterministic perturbed state, in terms of spread, bias and uncertainty as illustrated in Figure 12.

The distribution is wide and slightly skewed with the stochastic histogram having a large spread and the Gaussian fit having a large standard deviation for $z_{1}$. This is an indication of high sensitivity to stochastic fluctuations over the simulation interval, in line with the wide envelopes seen in the time-series analysis. The deterministic perturbed value is in the upper tail of the stochastic distribution, indicating that there is a systematic difference between the most probable stochastic values and the deterministic value. This offset is affected by nonlinear dynamics of the ensemble since the attractor geometry creates symmetry of the distribution of $z_{1}$ in the presence of noise.

The distribution of $z_{2}$ on the other hand is much more narrow and symmetric with a sharp peak and a smaller standard deviation. The deterministic perturbed value is in good agreement with the most probable value of the stochastic ensemble, and the ensemble is tightly concentrated around the mean, suggesting good agreement between the deterministic and stochastic predictions. This behavior is characteristic of the noise filtering property of the slow recovery subsystem, which is seen as a suppression of rapid stochastic fluctuations to statistical stability.

To summarize, the difference in the distribution of $z_{1}$ and $z_{2}$ shows that there are inherent differences between the sensitivity of the fast components of the PNS and the slow components. $z_{1}$ has a high variance and distributional skewness for the stochastic forcing, whereas $z_{2}$ is robust and is well clustered. This difference has important implications for the reliability and stability of neural dynamics in piezoelectric neuromorphic systems operating under realistic noisy conditions.

The basin of attraction analysis proves that the PNS is multistable with multiple attractors that coexist with each other in a large variety of initial conditions. This multistability is also facilitated by the analysis of the return map, which reveals smooth structures in weakly nonlinear regimes and multivalence behaviour at dynamical transitions, which suggests reorganization of Poincaré dynamics due to nonlinear mechanical-electrical coupling. This behavior, as seen through the lens of sensing, also suggests that the system can be used as a multi-state sensing element, with various attractors being associated with various mechanical input regimes. These nonlinear changes in return maps also imply that the system can be used in feature extraction and classification of states in piezoelectric sensing.

This coupling structure greatly exaggerates the effect of noise with stochastic forcing. Mechanical stimulation of the electrical subsystem by noise feeds back into the dynamics to cause stochastic dynamics between deterministic attractors and to converge to one long-term state with a probability distribution of possible asymptotic outcomes. This phenomenon is indicative of the inherent uncertainty presented by environmental variations in real-world piezoelectric sensing and neuromorphic systems where mechanical perturbation has a direct influence on the stability of electrical signals. Sensorly, this attractor switching can be viewed as a kind of signal modulation by noise which can increase sensitivity or decrease measurement reliability depending on the intensity of noise. This effect is well manifested in the trajectory-based comparative analysis and final-time statistical distributions which show that the mechanical-electrical feedback loop causes the long-term behavior of the PNS to be intrinsically sensitive to stochastic perturbations. This deterministic to probabilistic behavior is an important attribute of real world sensing devices that work in varying environmental conditions.

Additional structural data points to this noise sensitivity is retrieved by recurring analysis. Recurrence plots in the deterministic regime have long diagonal lines, which are in line with predictable quasi-periodic oscillations. With stochastic forcing, these diagonal structures are gradually destroyed and the recurrence texture becomes more and more irregular, which directly reflects the degradation of natural periodicity due to noise. Quantitatively, these structural changes can be realized by the reduction of recurrence determinism and the increase of recurrence entropy over the parameter sets that are studied. Thus, the degradation of recurrence structure can be directly linked to the loss of signal predictability in sensor outputs and RQA can be used to diagnose sensor performance in the presence of noise.

All in all, these findings indicate that the nonlinear mechanical-electrical interaction is the major determinant of the PNS dynamics, which determines its multistability, switching behavior caused by noise, and structure of the recurrence. The close match of the model predictions and observed dynamical responses is evidenced to support the proposed framework as a powerful diagnostic tool to evaluate the stability, reliability, and noise sensitivity of piezoelectric neuromorphic sensing devices under coupled electromechanical interactions. These results also indicate that the delicately adjusted stochasticity could be exploited to enhance sensitivity and adaptive behavior of the next-generation piezoelectric neuromorphic sensors.

## 6. Conclusions

In this study, a comprehensive analysis of the stochastic and deterministic forced dynamics of a system of piezoelectric neurons is provided. The study utilizes basin of attraction analysis, sensitivity to initial conditions, return map construction, RQA and statistical comparisons between different dynamical regimes to explore the stability, variability, and complexity of the system.

In the deterministic case, the system is well organized with clear quasi-periodic behavior and a toroidal structure of attractors. When the dynamics are moving away from the equilibrium, the system experiences more complex multistable dynamics near the boundaries of the attractors, which are less predictable. Finally, small changes in the initial conditions affect the transient solutions, although the attractor to which the system evolves is the same, demonstrating short-term sensitivity but long-term stability which is important for reliable sensing applications. In the case of stochastic forcing, the electromechanical coupling is enhanced and the response of the system is more variable. This leads to increased trajectory dispersion as well as tendencies toward attractor switching, and possible disruption of regular recurrence structures. There is an apparent separation of state variables, with the fast one having more fluctuations and wider statistical spreads and the slow one being comparatively stable and nearer to deterministic behavior.

This shows how slow dynamics can help to maintain coherence when there is noise. The results also reveal the usefulness of recurrence quantification measures for detecting changes in the dynamics that cannot be observed by analyzing time series or phase space data. A reduction in determinism and an increase in entropy with the noise intensity signals a decrease in predictability and increasing dynamical complexity, in particular. These measures offer a strong quantitative tool to describe stochastic effects in neuromorphic sensing systems. In general, the results show that system behavior is determined by the complex interplay between nonlinear electromechanical interactions, sensitivity to initial conditions and stochastic noise. This points to the potential use of the proposed model in enhancing the understanding and design of piezoelectric neuromorphic sensing systems in uncertain and noisy environments.

However, there are some drawbacks to be considered: The study is based on the idealized single-neuron model and simplified parameter and forcing assumptions, and may not fully reflect the complexities of real physical systems. The stochastic analysis is restricted to a Gaussian white noise, but other types of noise, for example coloured noise or multiplicative noise are not analyzed. Moreover, the present work is mostly numerical, and validation by experimentation is not provided, which might restrict the direct application.

Future work will involve its extension to networks of coupled piezoelectric neurons, experimental implementations of piezoelectric circuits and the incorporation of more realistic noise models. The extensions are likely to further improve the usability of the model for real-world neuromorphic sensory systems.

## Data Availability

Data is provided within the manuscript.
